# Functional role of delta and theta band oscillations for auditory feedback processing during vocal pitch motor control

**DOI:** 10.3389/fnins.2015.00109

**Published:** 2015-03-31

**Authors:** Roozbeh Behroozmand, Nadine Ibrahim, Oleg Korzyukov, Donald A. Robin, Charles R. Larson

**Affiliations:** ^1^Speech Neuroscience Lab, Department of Communication Sciences and Disorders, University of South CarolinaColumbia, SC, USA; ^2^Speech Physiology Lab, Department of Communication Sciences and Disorders, Northwestern UniversityEvanston, IL, USA; ^3^Departments of Neurology and Radiology, Research Imaging Institute, University of Texas Health Science Center San AntonioSan Antonio, TX, USA

**Keywords:** neural oscillation, sensory-motor integration, voice pitch control, vocalization, auditory feedback, absolute pitch, wavelet analysis, EEG

## Abstract

The answer to the question of how the brain incorporates sensory feedback and links it with motor function to achieve goal-directed movement during vocalization remains unclear. We investigated the mechanisms of voice pitch motor control by examining the spectro-temporal dynamics of EEG signals when non-musicians (NM), relative pitch (RP), and absolute pitch (AP) musicians maintained vocalizations of a vowel sound and received randomized ± 100 cents pitch-shift stimuli in their auditory feedback. We identified a phase-synchronized (evoked) fronto-central activation within the theta band (5–8 Hz) that temporally overlapped with compensatory vocal responses to pitch-shifted auditory feedback and was significantly stronger in RP and AP musicians compared with non-musicians. A second component involved a non-phase-synchronized (induced) frontal activation within the delta band (1–4 Hz) that emerged at approximately 1 s after the stimulus onset. The delta activation was significantly stronger in the NM compared with RP and AP groups and correlated with the pitch rebound error (PRE), indicating the degree to which subjects failed to re-adjust their voice pitch to baseline after the stimulus offset. We propose that the evoked theta is a neurophysiological marker of enhanced pitch processing in musicians and reflects mechanisms by which humans incorporate auditory feedback to control their voice pitch. We also suggest that the delta activation reflects adaptive neural processes by which vocal production errors are monitored and used to update the state of sensory-motor networks for driving subsequent vocal behaviors. This notion is corroborated by our findings showing that larger PREs were associated with greater delta band activity in the NM compared with RP and AP groups. These findings provide new insights into the neural mechanisms of auditory feedback processing for vocal pitch motor control.

## Introduction

The ability to control voice fundamental frequency (F0) is essential for animal vocalization and human speech. Humans dynamically change their voice F0 (perceived as pitch) in a variety of tasks for the purpose of vocal communication, singing and conveying behaviorally-relevant linguistic and emotional messages. The motor control of pitch during vocal production is a highly complex task that requires precise, simultaneous and coordinated movement of a large group of respiratory and laryngeal muscles in the peripheral vocal apparatus. The proposed state feedback control models (Zarate and Zatorre, 2005; Guenther et al., 2006; Tian and Poeppel, 2010, 2012a; Hickok et al., 2011; Houde and Nagarajan, 2011; Price et al., 2011; Hickok, 2012) suggest that vocalization control is mediated by feedforward mechanisms that use the efference copy (Wolpert et al., 2011) of vocal motor commands to generate internal predictions about sensory feedback associated with self-produced vocalizations. These internal predictions are hypothesized to be compared with the actual sensory feedback information, and the result of this comparative analysis has been proposed to drive subsequent vocal motor commands that control pitch during vocal production, singing or speaking.

The notion of feedforward and feedback integration mechanisms has been supported by evidence from studies that investigated behavioral vocal responses to pitch perturbation in voice auditory feedback. Findings of these studies have shown that humans control the pitch of their vocalizations by generating compensatory vocal responses that change their voice pitch in the opposite direction to pitch-shift stimuli in the auditory feedback (Larson, 1998; Chen et al., 2007; Liu and Larson, 2007; Behroozmand et al., 2012). These compensatory vocal mechanisms have been suggested to enable an individual to use online auditory feedback for vocal production and motor control.

Despite the significant progress in understanding the behavioral mechanisms of vocal pitch motor control, our knowledge about the underlying neural processes of this effect is relatively poor. Electrophysiological recordings of brain activity have revealed that the event-related potential (ERP) responses of electro-encephalography (EEG) signals and their magneto-encephalography (MEG) counterparts were suppressed at voice onset when the auditory feedback closely matched vocal production (no perturbation) during speaking compared with passive listening to its playback (Houde et al., 2002; Heinks-Maldonado et al., 2005, 2006; Behroozmand and Larson, 2011; Tian and Poeppel, 2013, 2012b; Kort et al., 2014). However, this motor-induced suppression effect was reduced when mismatches in timing and/or pitch frequency were introduced between internal feedforward predictions and auditory feedback (Tian and Poeppel, 2010, 2012a; Behroozmand and Larson, 2011; Behroozmand et al., 2011). Moreover, when pitch-shift stimuli were delivered in the middle of vocalizations, ERP responses to auditory feedback perturbations were enhanced during vocal production compared with playback (Behroozmand et al., 2009; Chen et al., 2013). This effect has been replicated and confirmed in more recent studies using invasive intracranial recordings of electro-corticography (ECoG) signals by showing that the power of the neural responses to pitch-shifted auditory feedback was enhanced within the high gamma frequency range (75–150 Hz) during vocalization compared with playback (Chang et al., 2013; Greenlee et al., 2013). A similar enhancement effect has also been observed in response to pitch-shifted vocalizations during single-unit recordings from the auditory cortex in Marmoset monkeys (Eliades and Wang, 2008), suggesting that humans and non-human primates may share common neural mechanisms for vocal production and motor control. These findings have been placed in the framework of a predictive coding model in which efference copies of the vocal motor commands suppress neural responses to predictable (unperturbed) auditory feedback. However, when auditory feedback is perturbed by a pitch-shift stimulus (unpredictable), neural responses are enhanced to detect and correct for production errors during vocalization and motor control.

A major shortcoming of previous electrophysiological studies of vocal pitch motor control is that they were limited to the analysis of ERPs and only allowed the study of mechanisms that are reflected by phase-synchronized (evoked) neural responses time-locked to the onset of pitch-shift stimuli. However, non-phase-synchronized (induced), and yet still event-related, changes in the power of ongoing neural activity may occur within specific frequency bands during performance of a task or in response to a stimulus (Crone et al., 2001). Compelling evidence from a number of studies have indicated that “induced” neural activities highlight functional activation of sensory-motor (Crone et al., 1998a,b), visual (Brunet et al., 2014), and auditory cortex (Mäkinen et al., 2005) in different experimental paradigms. More specifically and relevant to studies of auditory feedback perturbation, it has been proposed that induced neural response components of EEG within the delta (1–4 Hz) and theta (5–8 Hz) frequency bands reflect processes that are instantiated in response to the detection of novelty, conflict, and error in incoming sensory feedback stimuli (Cavanagh and Frank, 2014). These induced changes in the power of neural activity has been suggested to be an electrophysiological signature of neural processes that are activated in response to recognition of demand for increased top-down cognitive control (Cavanagh and Frank, 2014).

In the present study, we used data from a previously published work (Behroozmand et al., 2014) that examined behavioral and ERP responses to pitch-shifted voice auditory feedback in three groups of non-musicians (NM), relative pitch (RP), and absolute pitch (AP) musicians. In this study, we used the same dataset and applied a wavelet-based time-frequency analysis to expand our observations of neural responses to pitch-shifted voice auditory feedback in NM, RP, and AP groups. We followed a procedure proposed by Crone et al. (2001) wherein one can separately estimate evoked and induced neural response components from the EEG signals and study them independently using the time-frequency analysis technique. This procedure allowed us to examine band-specific modulation of evoked and induced power of the EEG signals in order to provide new insights into the unexplored aspects of neural mechanisms that incorporate auditory feedback to detect and correct for production errors during vocal pitch motor control.

An important advantage of comparing NM, RP, and AP groups in this experimental paradigm is that it allows us to examine how behavioral and neural mechanisms of pitch processing and motor control can be affected by musical training or the development of AP ability. It has been suggested that trained musicians and singers benefit from more robust feedforward mechanisms for processing and motor control of their voice pitch (Zarate and Zatorre, 2005, 2008; Zarate et al., 2010). More interestingly, musicians with AP have the unique ability to assign verbal labels to musical notes without the need to determine their tonal relationship to a reference (Takeuchi and Hulse, 1993). This extraordinary ability in AP musicians may arise from the development of specialized pitch processing mechanisms in their brain, which makes them an ideal model for studying the mechanisms of vocal pitch processing and motor control.

Based on the proposed functional significance of delta and theta band oscillations for conflict monitoring and feedback error processing (Cavanagh and Frank, 2014), we hypothesized that the spectro-temporal modulation of evoked and induced neural activity within these specific frequency bands would highlight new aspects of neural mechanisms involved in vocal pitch processing and motor control. Accordingly, we predicted that these neural response components would reflect new features of the sensory-motor mechanisms that incorporate auditory feedback for voice pitch error detection and motor control during vocal production. We also hypothesized that AP and RP musicians would exhibit a larger activation of evoked neural response components during vocal pitch error detection and motor control compared with the NM group. This hypothesis was supported by our previous findings showing that the amplitude of ERP responses to pitch-shifted auditory feedback was larger in AP and RP musicians compared with NMs (Behroozmand et al., 2014). Lastly, we hypothesized that the difference between behavioral measures of vocal pitch control in AP, RP, and NM groups would be correlated with modulation of evoked and/or induced neural responses to pitch perturbations in voice auditory feedback. These behavioral differences were examined in our previous study (Behroozmand et al., 2014) by showing that, as compared with the NM group, AP and RP musicians controlled their voice pitch better by generating faster compensatory vocal responses and were able to re-adjust and return to baseline pitch after feedback perturbation was removed. The potential for analysis of evoked and induced components of neural activity in the present study will shed light on novel aspects of neural mechanisms that underlie increased functional capacity of AP and RP musicians for vocal pitch processing and motor control.

## Materials and methods

### Subjects

The participants of this study included a sample of 34 subjects (15 male and 19 female, age range: 18–25 years) from Northwestern University with normal hearing and no history of neurological or speech disorders. In this population, 23 musician subjects were recruited from the Northwestern Bienen School of Music, while the remaining 11 untrained NMs were recruited from the general Northwestern population. Within the musician group, 12 subjects were RP and 11 subjects were AP musicians. A full description of subject recruitment criteria and verification of RP and AP ability in musicians was described in our previous study (Behroozmand et al., 2014). In summary, RP and AP possessors were first identified and recruited based on their self-identification, which were further confirmed using a battery of musical proficiency evaluation tests. These tests were administered in order to classify the ability of AP and RP musicians in pitch perception, identification, discrimination and production. The AP ability in musicians was determined based on their performance on specific modules of the tests which were designed to distinguish AP from RP ability in musicians. Subjects in the NM group also took this test despite a lack of musical training to function as a control. Musicians in the RP and AP groups were similar in terms of the onset age (mean 9.09 years in RP and 6.73 in AP) and duration of musical training (mean 11.64 years in RP and 12.23 in AP). All subjects in both groups started their musical training at the age of 11 or younger except for two subjects in the RP group that started music at the age of 14 and 16. Eight subjects in each RP and AP groups were formally trained vocalists and all of the remaining musicians in both groups had received vocal training at some point during their aural skills education.

### Experimental design

The experiment comprised two active vocalization blocks in which subjects were instructed to repeatedly produce and maintain a steady vocalization of the vowel sound /a/ at their conversational pitch and loudness. Despite the natural trial-by-trial variability during vocal production, subjects were asked to produce vocalization as consistently as possible across trials (i.e., with relatively similar pitch and loudness). During each vocalization, subjects maintained steady vowel productions for approximately 2–3 s while taking short breaks (2–3 s) between successive trials. Subjects were not asked to produce any voluntary vocal responses to the onset of pitch shift stimuli. Approximately, a total number of 240 vocalizations (120 per block) were produced and recorded during each block. During each vocalization trial, a brief (200 ms) pitch-shift stimulus perturbed the auditory feedback in the middle of vocalization with stimulus onset delay randomized between 500–1000 ms after the vocalization onset. The direction of stimuli was randomized between upward (+100 cents) and downward (−100 cents) pitch shifts in each trial. This led to the collection of approximately 120 vocalizations for each pitch-shift stimulus direction.

### Voice and EEG data acquisition

Technical details of data acquisition for this experiment are described in a previous publication (Behroozmand et al., 2014). To summarize, the experiment was conducted in a sound attenuated booth in which subject's voice and EEG signals were recorded during steady vowel sound vocalizations. The voice data were amplified with a Mackie mixer (model 1202-VLZ3), picked up using an AKG boomset microphone (model C420) and recorded at 10 kHz using PowerLab A/D Converter (Model ML880, AD Instruments). A custom-designed program in Max/Msp (Cycling 74, v.5.0) controlled an Eventide Eclipse Harmonizer to pitch shift the voice online and feed it back to the ears using Etymotic earphones (model ER1-14A). The Max/Msp program also generated TTL pulses to accurately mark the onset of pitch-shift stimuli in each trial. A 10 dB gain between voice and its feedback was maintained to partially mask air-born and bone-conducted voice feedback during vocalizations.

The EEG signals were recorded from 32 sites on the subject's scalp using an Ag–AgCl electrode cap (Easy-Cap GmbH, Germany) with an average reference montage. A BrainVision QuickAmp amplifier (Brain Products GmbH, Germany) on a computer utilizing BrainVision Recorder software (Brain Products GmbH, Germany) recorded the EEG signals at a 2 KHz sampling rate after applying a low-pass anti-aliasing filter with a 400 Hz cut-off frequency. The electro-oculogram (EOG) signals were simultaneously recorded using two pairs of bipolar electrodes to monitor eye blinks during the recording session.

### Analysis of vocal responses to pitch-shift stimuli

The pitch frequency of the recorded voice signals was extracted in Praat (Boersma and Weenink, 2001) using an autocorrelation method and then exported to MATLAB for further processing. The extracted pitch frequencies were segmented into epochs ranging from −200 ms before to 1500 ms after the onset of pitch-shift stimuli. Pitch frequencies were then converted from Hertz to the Cents scale to calculate vocal compensation in response to the pitch-shift stimulus using the following formula:

$$Vocal\;Compensation\;\left[cents\right]=1200\times log2\left(\frac{F}{F_{Baseline}}\right)$$

**Figure 1:**
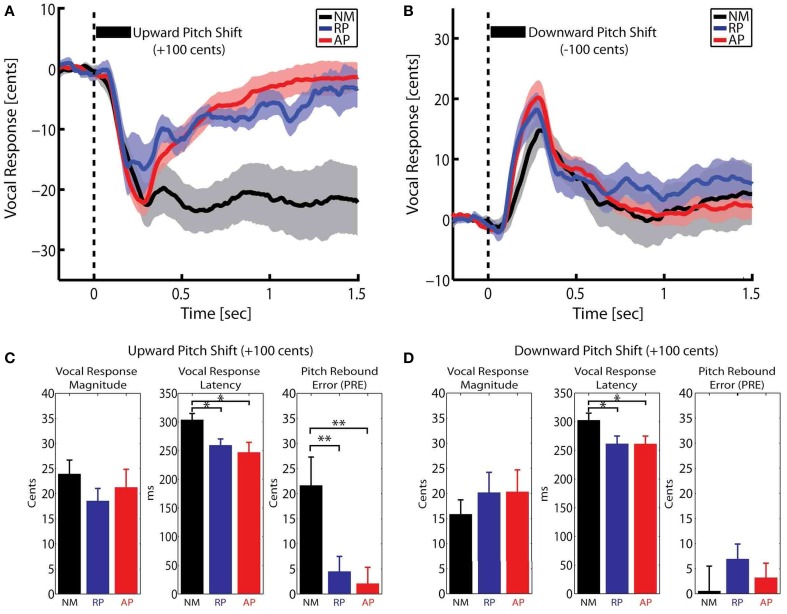
**Overlaid time course of the compensatory vocal responses to (A) upward and (B) downward pitch shift stimuli across three groups of non-musicians (NM) and relative pitch (RP) and absolute pitch (AP) musicians**. The bar plots in **(C,D)** show the extracted vocal response magnitude, latency and pitch rebound error (PRE) for upward and downward pitch shift stimuli, respectively. ^*^p < 0.05, ^**^p < 0.01.

Here, *F* is the post-stimulus pitch frequency and *F_Baseline_* is the baseline pitch frequency from −200 to 0 ms pre-stimulus. The calculated pitch contours in Cents were averaged across NM, RP and AP groups for upward and downward stimuli separately. Figures 1A,B show the overlaid vocal responses across all three groups for upward and downward pitch-shift stimuli, respectively. The magnitude and latency of vocal responses were extracted for the most prominent peak in a time window from 0–400 ms post-stimulus. Moreover, a new measure of *“Pitch Rebound Error (PRE)”* was extracted as the absolute value of the difference between the mean of post-stimulus vocal responses at latencies from 1000–1500 ms and the pre-stimulus baseline pitch from −200 to 0 ms. The PRE measure was extracted as a behavioral marker to determine the degree by which the subjects had the ability to re-adjust their voice pitch and return it back to the baseline level after the offset of the pitch-shift stimulus.

### Extracting evoked and induced neural responses

**Figure 2:**
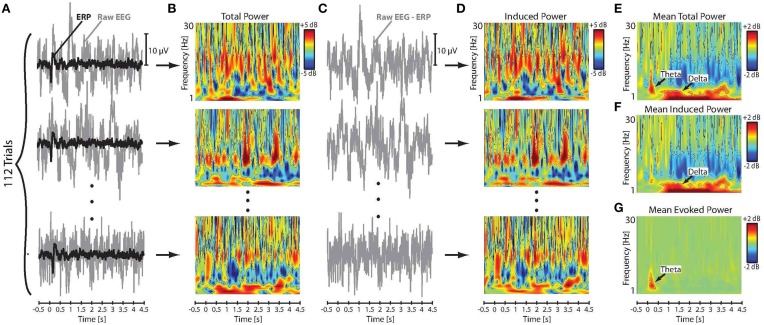
**(A)** Time series of the raw EEG signal and the extracted ERP responses to downward pitch shift stimuli across 112 trials for an example AP musician subject. **(B)** Time-frequency maps of the log-transformed total power (evoked and induced) of the single-trial raw EEG signals. **(C)** Time series of the single-trial induced neural responses calculated by subtracting ERPs from the raw EEG signals. **(D)** Time-frequency maps of the single-trial log-transformed power of the induced neural activity in response to pitch shift stimuli. Mean of the across-trial **(E)** total, **(F)** induced, and **(G)** evoked power in response to pitch shift stimuli.

We followed an approach introduced by Crone et al. (2001) to extract the evoked (phase-synchronized) and induced (non-phase-synchronized) components of the event-related neural activity from the EEG signal. In this method, the raw EEG time series were first segmented into trial epochs ranging from −500 ms before to 4500 ms after the stimulus onset. Following segmentation, trials in which the amplitude of the EEG and/or EOG signals exceeded ± 50 μV were excluded from analysis in order to reject the effect of artifact due to movement and eye blinks. Baseline correction was then applied to each individual trial by subtracting the mean of the pre-stimulus amplitude at −500–0 ms from all data points in the corresponding epoch. Individual trials were then averaged separately for each subject and stimulus direction to obtain the evoked ERP responses to pitch-shift stimuli. Subsequently, the extracted ERPs were subtracted from the raw EEG signal on a trial-by-trial basis to calculate the induced component of neural responses to pitch-shift stimuli. Figures 2A,C show a summary of this procedure for an example AP musician subject. It has been argued by Crone et al. (2001) that this procedure minimizes the contribution of the evoked neural activity and yields a reliable estimate of the induced component in response to a desired stimulus. Given a sufficient number of trials, this technique is computationally equivalent to the previously proposed inter-trial variance method for extracting induced neural responses from the EEG signal (Kalcher and Pfurtscheller, 1995).

### Time-frequency analysis of EEG

Time-frequency analysis was performed by applying a complex Morlet wavelet transform with center frequencies ranging from 1 to 30 Hz (1 Hz resolution) to the raw EEG and the extracted induced neural activity on a trial-by-trial basis. The choice of this frequency range was based on the results of our preliminary analysis that showed no significant post-stimulus changes in the spectral power of the EEG signals at frequencies above 30 Hz. In this analysis, the wavelet constant ratio was defined as f_c_/σ_f_ = 4, where f_c_ is the center frequency of the wavelet and σ_f_ is its standard deviation in the frequency domain defined as σ_f_ = 1/(2Ψ_t_). As an example, for a theta band component at 5 Hz, this leads to a wavelet function with 254.6 ms width in time (2σ_t_) and a spectral bandwidth (2σ_f_) of 2.5 Hz. The power of the event-related neural responses to the pitch-shift stimulus was calculated by taking the absolute value of the sum of the squared complex wavelet coefficients for the raw EEG and extracted induced neural activity on a trial-by-trial basis. For each individual trial, changes in signal power in response to pitch-shift stimuli were calculated for each frequency using the log transform of the ratio between post-stimulus response power (P_Response_) normalized to baseline power (P_Baseline_) in a time window from −500–0 ms pre-stimulus according to the following formula:

$$Power\left[dB\right]=10\times log10\left(\frac{P_{Response}}{P_{Baseline}}\right)$$

The log transformation function was used to ensure that the data were normally distributed for statistical analysis. Figures 2B,D show the calculated time-frequency maps of single-trial power changes in the raw EEG (total power: evoked and induced) and the induced neural activity in response to pitch-shift stimuli for an example AP musician subject. The mean of the total and induced power changes in response to pitch-shift stimuli was calculated for each subject by averaging log-transformed power changes in the raw EEG and induced neural activity across all trials, respectively. Figures 2E,F show the time-frequency maps of the mean of the total and induced power changes elicited by pitch shift stimuli. Since the raw EEG signal incudes both evoked and induced neural response components, the mean power changes in the evoked neural responses was calculated by subtracting the induced power from the power of the raw EEG signal on a trial-by-trial basis and then averaging the resulting power across all trials for each subject (Figure 2G). Results of our preliminary analysis confirmed that extracting the power of the evoked neural response components using this procedure was identical to applying wavelet transformation directly to the ERPs in response to pitch shift stimuli in voice auditory feedback. Therefore, in the present study, we report evoked responses extracted using the former method in which induced power of neural activity was subtracted from the total power (evoked + induced) of the raw EEG signals. Figure 2E shows the time-frequency map of the evoked power in responses to pitch-shift stimuli in an example AP musician subject.

## Results

### Duration of vocal production

Trial-by-trial durations of vocalizations and breaks (silent time periods between successive vocalizations) were calculated across NM, RP, and AP groups for upward and downward pitch-shift stimuli, separately. Results of the analysis using a Mixed-ANOVA model with groups as the between-subject factor and stimulus direction as the within-subject factor (repeated measure) indicated no significant difference between the duration of vocalizations and breaks across subject groups and/or stimulus directions. For upward pitch-shift trials, the mean duration of vocalizations was 2.64 s (std: 0.23), 2.91 s (std: 0.31), and 2.77 s (std: 0.33) and the mean duration of breaks were 2.46 s (std: 0.4), 2.63 s (std: 0.4), and 2.50 s (std: 0.29) for NM, RP, and AP groups, respectively. For downward pitch-shift trials, the mean duration of vocalizations were 2.80 s (std: 0.27), 2.92 s (std: 0.30), and 2.87 s (std: 0.34) and the mean duration of breaks were 2.48 s (std: 0.42), 2.62 s (std: 0.39), and 2.49 s (std: 0.29) for NM, RP and AP groups, respectively. The mean of the inter-vocalization interval (time period between the onset of successive vocalizations), averaged across subject groups and stimulus directions, was 5.33 s (std: 0.34).

### Vocal responses to auditory feedback pitch perturbation

A Mixed-ANOVA model with groups (NM, RP, and AP) as the between-subject factor and stimulus direction (upward vs. downward) as the within-subject factor (repeated-measure) was used to analyze vocal response magnitude, latency and pitch rebound error (PRE). Bar plots in Figures 1C,D summarize the results of this analysis. For vocal response peak magnitudes, results did not show any significant effect. For vocal response peak latencies, results showed a significant main effect of group [*F*_(2, 31)_ = 4.69, *p* < 0.05]. *Post-hoc* tests using Bonferroni's correction revealed that the vocal response latencies were significantly longer in NM compared with RP and AP groups for both upward (*p* < 0.05) and downward (*p* < 0.05) pitch-shift stimuli. However, no significant difference was found between the latencies of the vocal responses in RP vs. AP groups. For the PRE measure, results showed a significant main effect of stimulus direction [*F*_(1, 31)_ = 12.02, *p* < 0.01] and a significant group × stimulus direction interaction [*F*_(2, 31)_ = 9.86, *p* < 0.01]. *Post-hoc* tests using Bonferroni's correction revealed that the significant main effect of stimulus direction was only due to a significantly larger PRE (*p* < 0.01) in the NM group in response to upward compared with downward pitch-shift stimuli. Moreover, PREs in response to upward pitch-shift stimuli were found to be significantly larger (*p* < 0.01) in the NM compared with RP and AP groups. There was no significant difference of PREs between RP and AP groups.

### Neural responses to auditory feedback pitch perturbation

**Figure 3:**
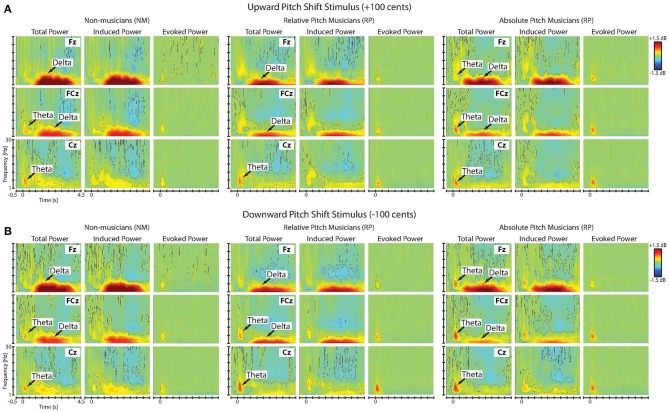
**Time-frequency maps of the grand-averaged total, induced and evoked power of the neural responses to (A) upward and (B) downward pitch shift stimuli across non-musicians (NM), relative pitch (RP), and absolute pitch (AP) musicians**.

Figures 3A,B show the time-frequency maps of the grand-averaged total, induced and evoked power in NM, RP, and AP groups in responses to upward and downward pitch-shift stimuli, separately. As can be seen in this figure, the onset of the pitch-shift stimulus elicited two distinctly different neural response components that could be differentiated based on their latency, duration, spectral bandwidth and response type (evoked vs. induced). The first component appeared as an evoked neural response with a power increase within the theta frequency band (5–8 Hz) that was predominantly present in the time-frequency map of the total and evoked but not the induced power responses (Figure 3). This evoked theta band activity had an across-group mean onset latency of 41 ms (std: 3.29 ms), peaked at 202 ms (std: 7.03 ms), and lasted for 526 ms (std: 14.12 ms) following upward and downward pitch-shift stimulus onset. The second component appeared as an induced neural response with power increase within the delta frequency band (1–4 Hz) that was almost equally present in the time-frequency map of both the total and induced but not the evoked power responses. This induced delta band activity had an across-group mean onset latency of 1050 ms (std: 44.17 ms), peaked at 1806 ms (std: 16.55 ms), and lasted for 2815 ms (std: 39.33 ms) following upward and downward pitch-shift stimulus onset.

In order to analyze these data, the power of the evoked theta band (5–8 Hz) and induced delta band (1–4 Hz) neural activity were separately extracted for each subject and stimulus direction for 18 electrode locations (F3, Fz, F4, FC3, FCz, FC4, C3, Cz, C4, CP3, CPz, CP4, P3, Pz, P4, PO3, POz, and PO4). The power of the evoked theta and induced delta band activity were extracted within a 50 ms time window closely centered near the response peaks at 200 and 1800 ms post-stimulus latencies, respectively. A Mixed-ANOVA model with groups as the between-subjects factor and stimulus direction (upward vs. downward), frontality (Frontal: F3, Fz, F4, Fronto-central: FC3, FCz, FC4, Central: C3, Cz, C4, Centro-parietal: CP3, CPz, CP4, Parietal: P3, Pz, P4, and Parieto-occipital: PO3, POz, PO4), and laterality (Left: F3, FC3, C3, CP3, P3, PO3, Right: F4, FC4, C4, CP4, P4, PO4, and Center: Fz, FCz, Cz, CPz, Pz, POz) as the within-subjects factors (repeated measures) were used to analyze the power of evoked theta and induced delta band responses to pitch-shift stimuli.

**Figure 4:**
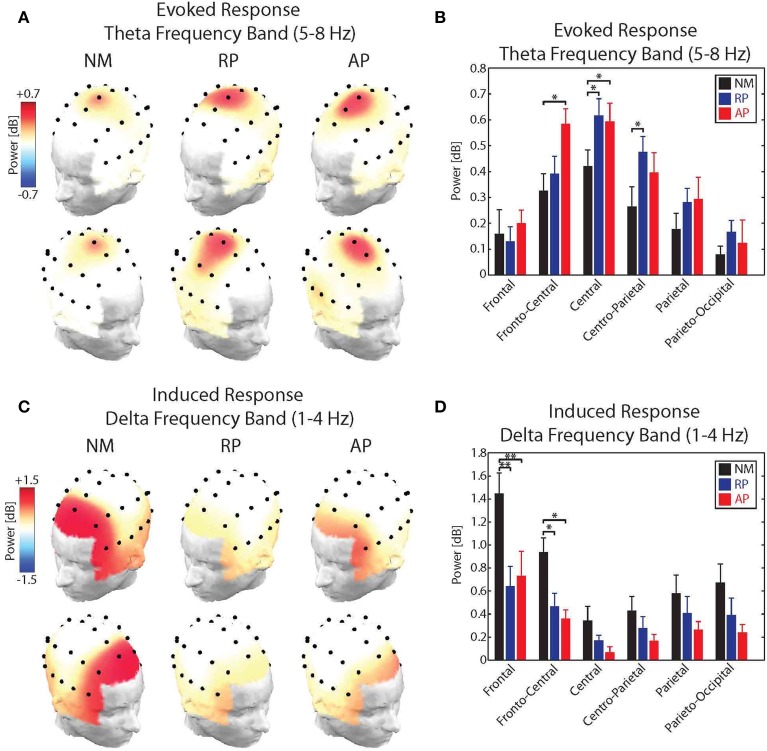
**(A)** Topographical distribution maps of the power of the evoked theta band (5–8 Hz) activity in response to upward and downward pitch shift stimuli across non-musicians (NM), relative pitch (RP), and absolute pitch (AP) musicians. **(B)** Bar plots representation of the power of the evoked theta band activity for frontal, fronto-central, central, centro-parietal, parietal, and parieto-occipital electrode locations across non-musicians (NM), relative pitch (RP), and absolute pitch (AP) musicians. **(C)** Topographical distribution maps of the power of the induced delta band (1–4 Hz) activity in response to upward and downward pitch shift stimuli across non-musicians (NM), relative pitch (RP), and absolute pitch (AP) musicians. **(D)** Bar plots representation of the power of the induced delta band activity for frontal, fronto-central, central, centro-parietal, parietal, and parieto-occipital electrode locations across non-musicians (NM), relative pitch (RP), and absolute pitch (AP) musicians. ^*^p < 0.05, ^**^p < 0.01.

For the evoked theta band responses, results of the analysis showed significant main effects of group [*F*_(2, 31)_ = 5.91, *p* < 0.05] and Frontality [*F*_(5, 155)_ = 18.13, *p* < 0.001] as well as a significant group × frontality interaction [*F*_(10, 155)_ = 2.56, *p* < 0.01]. No effect of stimulus direction or laterality was found for the power of the evoked theta band responses to pitch-shift stimuli. *Post-hoc* test using Bonferroni's correction revealed that the evoked theta band power in response to pitch-shift stimuli had a fronto-central distribution with the strongest responses at the FCz and Cz electrodes (*p* < 0.01). The topographical distribution maps of the evoked theta band power is shown in Figure 4A. In addition, we found that the power of the evoked theta band activity was significantly stronger in the AP compared with NM group at the FCz and Cz electrodes (*p* < 0.05) and in the RP compared with the NM group at Cz and CPz electrodes (*p* < 0.05). However, no significant difference in the power of the evoked theta band power was found between AP and RP musician groups. The result of this analysis is summarized in the bar plots shown in Figure 4B.

For the induced delta band responses, results of the analysis showed significant main effects of group [*F*_(2, 31)_ = 12.24, *p* < 0.01] and Frontality [*F*_(5, 155)_ = 32.45, *p* < 0.001] as well as a significant group × frontality interaction [*F*_(10, 155)_ = 6.83, *p* < 0.01]. No effect of stimulus direction or laterality was found for the power of the induced delta band responses to pitch-shift stimuli. *Post-hoc* test using Bonferroni's correction revealed that the induced delta band power had a frontal distribution with the strongest responses at the Fz electrode (*p* < 0.01). The topographical distribution maps of the induced delta band power is shown in Figure 4C. In addition, we found that the power of the induced delta band activity was significantly stronger in the NM compared with RP and AP groups at the Fz (*p* < 0.01) and FCz (*p* < 0.05) electrodes. However, no significant difference in the power of the induced delta band power was found between AP and RP musician groups. The result of this analysis is summarized in the bar plots shown in Figure 4D.

### Correlation analysis

**Figure 5:**
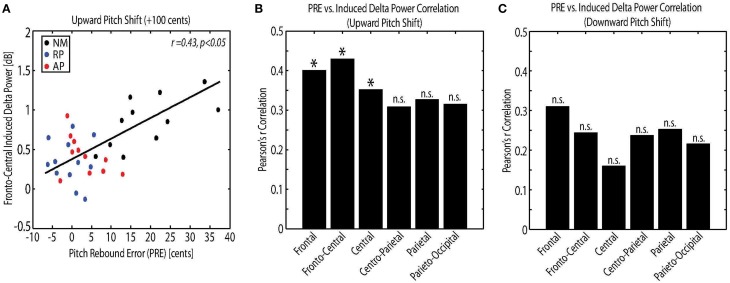
**(A)** Correlation analysis between the pitch rebound error (PRE) and the power of the fronto-central induced delta band activity in response to upward pitch shift stimulus across non-musicians (NM), relative pitch (RP), and absolute pitch (AP) musicians. Bar plot representation of the correlation analysis between the pitch rebound error (PRE) and the power of the frontal, fronto-central, central, centro-parietal, parietal and parieto-occipital induced delta band activity in response to **(B)** upward and **(C)** downward pitch shift stimulus across non-musicians (NM), relative pitch (RP), and absolute pitch (AP) musicians. ^*^p < 0.05.

The relations among measures of behavioral vocal responses and neural activity in response pitch-shift stimuli were examined using Pearson's correlation analysis. For this analysis, the power of the evoked theta and induced delta band activity were used as the neural correlates, and the magnitude, latency and PRE of vocal responses were used as the behavioral correlates. Results showed a significant correlation between the PRE and the power of the induced delta band activity at frontal (*r* = 0.40, *p* < 0.05), fronto-central (*r* = 0.43, *p* < 0.05) and central (*r* = 0.35, *p* < 0.05) electrode locations only for upward pitch-shift stimulus. Figure 5A shows an example of this correlation analysis between PRE and fronto-central induced delta band power in response to upward pitch-shift stimulus. The result of PRE and induced delta correlation at different electrode locations is summarized for upward and downward pitch-shift stimuli in Figures 5B,C, respectively. No significant correlation was found between the measures of neural activity and the magnitude and latency of the behavioral vocal responses to pitch-shift stimuli.

## Discussion

The present study utilized a novel approach to understand the neural bases of auditory feedback processing during vocal production and motor control. We used a wavelet-based method for time-frequency analysis of the EEG signals in order to examine the functional role of cortical neural oscillations for vocal pitch motor control in response to perturbations in the auditory feedback. This analysis expanded our observations from a previously published study in which the measures of ERPs were extracted and analyzed for the same data set used in the present study (Behroozmand et al., 2014). In that previous study (Behroozmand et al., 2014), results showed that the N1 component of ERPs in response to pitch-shift stimuli was significantly stronger in the right hemisphere for both AP and RP musicians compared with the NM group. In addition, we found that the P2 component of ERPs in the left hemisphere was significantly stronger in AP and RP musicians compared with NMs, and also was stronger for AP compared with RP musicians. Moreover, we found that the latency of compensatory vocal responses to pitch shifts was correlated with the amplitude of the P2 component, with larger P2 responses related to faster vocal compensation of pitch in AP and RP musicians compared with NMs. These findings suggested that, as compared with NMs, AP, and RP musicians use enhanced right-hemisphere mechanisms for auditory processing of pitch changes and use enhanced left-hemisphere mechanisms for voice pitch motor control in response to perturbations in the auditory feedback. Stronger P2 responses in AP compared with RP musicians in the left hemisphere also suggested that this specific ERP component is a neurophysiological marker of enhanced pitch processing in musicians with AP ability. In a follow-up study by Parkinson et al. (2014), it has been suggested that the enhanced pitch processing ability in AP musicians may be driven by the difference in effective connectivity between sensory-motor networks of left and right hemispheres.

As compared with the ERPs, time-frequency analyses of brain activity provided new insights into neural mechanisms of vocal pitch motor control by highlighting processes that were reflected by distinctly different patterns of neural oscillations elicited in response to auditory feedback perturbations. In the present study, these neural oscillations were identified by an increase in the power of the phase-synchronized (evoked) theta band (5–8 Hz) and non-phase-synchronized (induced) delta band (1–4 Hz) activity in response to pitch-shift stimuli during vocal motor control.

Results of our analysis revealed that the evoked theta band activity appeared as a neural response component that followed pitch-shift stimulus onset with short latencies (~40 ms) and lasted for approximately 500 ms post-stimulus. We found that the power of the evoked theta band activity at 200 ms had a fronto-central distribution and in some ways mimicked the properties of the P2 ERP responses in our previous study (Behroozmand et al., 2014) by the fact that it was stronger in AP and RP musicians compared with NMs. However, the evoked theta band activity differed from the previously reported P2 ERP responses (Behroozmand et al., 2014) by the fact that it was not lateralized and was not correlated with the peak latency of the compensatory behavioral vocal responses. However, based on the overlap between the time course of theta activity and behavioral vocal responses, we suggest that these neural oscillations may partially be accounted for by the mechanisms that facilitate sensory-motor processes of pitch change detection and correction in voice auditory feedback. Moreover, increased theta band power in RP and AP musicians compared with NMs suggests that this neural oscillation component is a neurophysiological marker of enhanced cognitive and perceptual ability for vocal pitch processing in RP and AP musicians.

The inconsistency in some aspects of the evoked theta band activity in this study and P2 ERP responses in our previous study (Behroozmand et al., 2014) may arise from the fact that the power increase within the theta band may not be directly related to modulation of a specific ERP component (e.g., P2) in response to pitch-shift stimuli. This difference is dictated by inherent properties of wavelet transformation in which, for a given point in time, the spectral power within different frequency bands is calculated using Morlet functions with variable durations and spectral bandwidths (please see methods for more details). As mentioned earlier, with the wavelet constant ratio specified for data analysis in this study, the duration of the Morlet function for a theta band component at 5 Hz is 254.6 ms. This means that the extracted theta band power at 200 ms does not directly relate to the P2 ERP component at the same latency, but rather may receive contributions from other ERP components at shorter and longer latencies. In other words, the extracted evoked activity within the theta band reflects an overall change in the power of low-frequency oscillations at approximately 5–8 Hz and can encompass multiple ERP components. In wavelet analysis, this effect is referred to as “smearing” and is analogous to a “windowing” effect in traditional Fourier transformation for spectral analysis. Therefore, it is important to take into account that a direct point-to-point relationship may not necessarily exist between the profile of the evoked power and ERP responses to pitch-shift stimuli. Based on these reasons, a direct comparison between changes in power of the evoked theta and ERP response components may not be strongly justified.

The specific functional role associated with neural oscillations in the frontal cortex as measured by human EEG is not well understood. A number of studies have suggested that frontal oscillations within the theta frequency band (5–8 Hz), recorded from EEG electrodes over pre-frontal cortex (PFC), reflect neural processes that are instantiated in response to the detection of novelty, conflict, punishment, and error in incoming sensory feedback stimuli. The elicited frontal theta band activity in response to these phenomena is an electrophysiological signature of common neural processes that are activated in response to recognition of demand for increased top-down cognitive control (Cavanagh and Frank, 2014). The large-amplitude low-frequency theta band oscillations have broad distributions across different cortical and sub-cortical networks (Raghavachari et al., 2006) and can be used to execute cognitive control by orchestrating communication between multiple neuronal assemblies in spatially-segregated brain regions. Converging evidence from the results of source localization on EEG signals have suggested that the neural substrates involved in generation of frontal theta activity comprise areas within anterior (ACC) and middle cingulate cortex (MCC) as well as the supplemental motor areas (SMA) (Debener et al., 2005; Hauser et al., 2014). Evidence from recent functional neuroimaging studies has provided support for the role of these brain areas in production and motor control of pitch during vocalization, speaking and singing (Tourville et al., 2008; Zarate and Zatorre, 2008; Zarate et al., 2010; Parkinson et al., 2012; Zheng et al., 2013; Behroozmand et al., 2015). The ACC and MCC are anatomically connected with pre-frontal, parietal and motor cortices and are central stations for processing multi-modality sensory stimuli and assigning control over other brain areas in tasks that require error detection and conflict monitoring (Liu et al., 2013). The SMA receives input from different sensory-motor areas and is a major contributor to direct control of movement (Rosso et al., 2014). The anatomical connectivity and functional significance of the proposed frontal theta generators (ACC, MCC, and SMA) suggests that this neural oscillation component is a neurophysiological marker of cognitive control for driving subsequent motor behaviors in tasks that require error detection and correction.

The theta band activity in response to auditory feedback pitch perturbation in the present study share a common spectral signature and topographical distribution with theta activations recorded in earlier studies of cognitive control (Cavanagh and Frank, 2014). Based on this evidence, we propose that the evoked activity within theta band in the present study reflects top-down mechanisms by which humans incorporate auditory feedback to monitor and control their voice pitch during speech. At the central nervous system level, this process requires the brain to orchestrate neural activity between a large group of functionally-related neuronal populations involved in sensory-motor and cognitive processing of pitch during vocalization.

The top-down coordination of these networks is geared toward continuous monitoring of sensory (i.e., auditory) feedback information for error detection and correction during vocal production. Due to the inherent complexity of speech production and motor control, fine coordination of neural activity across sensory-motor and cognitive networks seems necessary to achieve goals of such complex motor tasks during speaking. The evoked theta activity appears to be a compelling candidate mechanism to provide a temporal window for functionally-relevant communication between neural networks involved in auditory feedback processing for speech motor control. Our data show that the time range of the evoked theta activity overlaps with the temporal window within which behavioral vocal compensations were elicited in response to pitch perturbation in the auditory feedback. Therefore, we suggest that the frontal theta band activity highlights neural mechanisms that are involved in vocal pitch monitoring and control and serves as a neurophysiological marker of training-induced enhanced cognitive and perceptual ability for pitch processing in RP and AP musicians.

It has been suggested that the delta and theta oscillations reflect neural processes that underlie relatively similar functions during the performance of various cognitive tasks (Bas and Schurmann, 2001). In addition, low-frequency rhythms within the delta frequency band have been suggested to provide a general oscillatory framework for sensory feedback processing of temporally-predictable stimuli that are generated as a result of biologically-relevant movements such as vocal communication (Schroeder et al., 2008). Results of our time-frequency analysis revealed that the obtained delta band activation was an event-related increase in the power of induced neural response components at 1–4 Hz with a consistent spectro-temporal profile of activation across all subject groups (NM, RP, and AP) and stimulus directions (upward vs. downward). Although the delta activity had a frontal scalp distribution, due to the following reasons, we believe that it is unlikely that this neural response component may have been contaminated by eye blinks or other movement-related artifacts. First, as opposed to temporally-variable muscle artifact (e.g., random eye blinks or movement that are not time-locked to stimulus onset), the delta band activity was an induced neural response component that was time-locked to the onset of pitch-shift stimulus and occurred, with a relatively consistent temporal profile, at about 1 s and lasted for approximately 3 s after the onset of pitch-shift stimulus. Second, similar to evoked theta band activity, the induced delta neural response component was identified as the result of the carefully-implemented and well-controlled analysis procedures that used standard signal processing methods to remove the effect of muscle artifact from the EEG signals prior to time-frequency analysis of neural activity (for more detail please see Materials and Methods Section). Furthermore, by comparing the duration of the delta band activity relative to the duration of inter-vocalization intervals, it can be claimed that the extracted delta band power within each trial is not influenced by the onset of subsequent vocalizations in the next trial.

Results of our analysis indicated that the induced delta band activity extended beyond the duration of vocalizations and it was correlated with the behavioral measures of PRE during rebound period after the pitch-shift stimulus was removed. In addition, as opposed to evoked theta, the time course of the induced delta band activity did not overlap with compensatory vocal responses to pitch shifts in voice auditory feedback. Although one possible explanation for the extension of delta beyond vocalizations might be due to smearing effect of wavelet analysis, our findings suggest that the underlying neural mechanisms of induced delta are independent of the motor act of vocalization and subserve other behaviorally-relevant functions during vocal pitch motor control. We propose that the delta activation may reflect motor adaption neural processes that update the current state of the sensory-motor representation during vocal production and motor control. These adaptive neural process are potentially involved in monitoring production errors to evaluate behavioral performance after feedback perturbation is eliminated, and can give rise to the emergence of motor behaviors that enhance effective communication in unpredictable future environments. This notion is supported by our findings showing that larger increases in delta band power were correlated with greater PREs in response to upward pitch-shift stimuli in NMs compared with AP and RP musicians. Larger increases in the delta power for greater PREs in NMs might be an indication of assigning neural resources that are required to update the internal representation of the vocal pitch based on the most recent history of the auditory feedback information during vocal production. Based on this notion, AP and RP musicians supposedly benefit from a more robust internal feedforward representation and are less sensitive to auditory feedback perturbations for regulating their voice pitch, as indicated by smaller degrees of PREs and delta band power increases after the pitch-shift stimuli are removed. This proposed functional mechanism of delta band activity highlights fundamental differences between neural processes of vocal pitch motor control in AP and RP musicians compared with the NM group.

As discussed in our previous study (Behroozmand et al., 2014), we proposed that the observed difference in PREs between upward and downward stimuli in NM may potentially be attributed to the difference in the pattern of intrinsic laryngeal muscle activations (e.g., cricothyroid and thyroarytenoid) during vocal compensation (Liu et al., 2011). In order to decrease voice pitch in response to upward pitch-shift stimuli (vocal compensation), the vocal motor system needs to reduce the tension of the vocal folds by relaxing laryngeal muscles, and then contract them during rebound period (after perturbation is removed) to re-adjust voice pitch to baseline. However, for downward pitch shifts, the vocal motor system must increase laryngeal muscle activation during the compensation period and relax them during rebound. According to this pattern of activation, rebound in response to upward pitch-shift stimuli is facilitated by an active motor process that increases laryngeal muscle tension, whereas for downward stimuli, rebound is facilitated by a passive muscle relaxation process. This difference in the activation pattern of laryngeal muscles in conjunction with our findings related to behavioral vocal responses to pitch shifts highlighted fundamental differences between the underlying neural processes of vocal pitch motor control between NM, RP, and RP groups. Our data suggest that both AP and RP musicians benefit from a robust feedforward representation of their voice pitch and are capable of actively recruiting motor functions to re-adjust pitch to baseline during rebound. However, the NM group were only able to re-adjust their pitch (small PREs) in response to downward stimuli when rebound required a passive motor process for muscle relaxation, and they showed large PREs in response to upward pitch shifts in voice auditory feedback. By combining the results of these behavioral differences with neural responses obtained using time-frequency analysis, our study suggests that the induced delta activation is a neurophysiological marker of enhanced pitch motor control mechanisms in musicians compared with non-musicians. As mentioned earlier, this notion is corroborated by our findings showing that the power of the induced delta component was significantly correlated with the degree of PRE in the NM compared with AP and RP groups.

It is worthwhile to mention that, compared with similar previous studies (Houde et al., 2002; Behroozmand et al., 2009; Chang et al., 2013; Greenlee et al., 2013), a shortcoming of the present study was the absence of the playback condition. The inclusion of the playback condition and comparing its results with speaking could potentially have additional benefits because it would have allowed us to study sensory and motor mechanisms of voice pitch motor control independently across NM, RP, and AP groups. In the present study, we were limited by reporting findings of behavioral and band-specific neural components in response to pitch-shift stimuli only during vocalization and comparing them across NM, RP, and AP groups. Therefore, the absence of the playback condition precluded us from determining the independent contribution of sensory and motor mechanisms in generating the observed delta and theta band oscillations during vocal pitch control. Although our findings indicate that the evoked and induced neural response components reflect different aspects of pitch error detection and motor control mechanisms in NM, RP, and AP groups, future studies will be needed to answer the question of which of the above oscillatory mechanisms are accounted for by the sensory and/or motor mechanisms during vocal pitch control. We propose that the theta mechanisms may partially be involved in sensory processes of feedback error detection that subsequently drives motor control. However, based on the results of our correlation analysis, we suggest that the theta oscillations may not directly reflect vocal motor activity for vocal compensation. This suggestion is based on the fact that the evoked theta power was not found to be correlated with behavioral measures of compensatory responses. However, delta oscillations were correlated with behavioral measures of PRE, suggesting that they may be accounted for by adaptive motor processes that update the state of sensory-motor networks for driving subsequent vocal behaviors for future vocal control tasks. This notion is corroborated by our findings showing that larger degrees of PRE in NM group was correlated with stronger activation of theta band induced oscillations in this group compared with AP and RP musicians.

### Conflict of interest statement

The authors declare that the research was conducted in the absence of any commercial or financial relationships that could be construed as a potential conflict of interest.
